# Effect of drying methods and blending ratios on dough rheological properties, physical and sensory properties of wheat–taro flour composite bread

**DOI:** 10.1002/fsn3.444

**Published:** 2016-12-02

**Authors:** Gidmwork Abera, W.K. Solomon, Geremew Bultosa

**Affiliations:** ^1^ Department of Food Science and Postharvest TechnologyAdama Science and Technology UniversityAsselaEthiopia; ^2^ Department of Food and Nutrition Sciences Faculty of Consumer SciencesUniversity of SwazilandLuyengoSwaziland; ^3^ Department of Food Science and TechnologyBotswana University of Agriculture and Natural ResourcesGaboroneBotswana

**Keywords:** drying, farinographic properties, mixing tolerance index, sensory properties, taro flour

## Abstract

The study was conducted to evaluate the effect of taro drying methods and blending ratios on the physical quality attributes and sensory quality of wheat–taro bread and rheological properties of the blend dough. Farinographic properties like water absorption capacity, dough development time, dough stability time, time to break down, mixing tolerance index, and farinographic quality number were significantly (*p* < .05) affected by drying methods and blending ratio and their interaction. Increased taro flour (10–20 g) per 100 g of wheat flour resulted in an increased water absorption capacity (57.38%–58.23%) and mixing tolerance index (67.33–70.21 FU). The sensory analysis had revealed that as taro flour blending ratio increased the acceptability of blended breads were reduced. With respect to physical and sensory properties, the control bread had better acceptability than that of 10, 15, and 20 g taro flour‐mixed bread. The study revealed that there is possibility of incorporating taro flour up to 15 g per 100 g of wheat flour with acceptable sensory attributes of the composite bread.

## 1 Introduction

Bread is a carbohydrate‐rich source of starch and dietary calories, and hence is an important part of a balanced diet. The major or mandatory ingredients in bread making are flour, water, salt, and yeast. However, due to the high cost, geographical scarcity, and high demand of wheat flour, efforts are being directed toward the provision of alternative source of flour. Because of this, cocoyam, cassava, taro, and other root and tubers crops have been found to be additional ingredients of major raw materials for bread making.

Taro (*Colocasia esculenta* (L.) Schott) is a major tuber crop cultivated in the tropical and subtropical regions of the world. The world average production of taro is about 6.2 tons/ha while African average is 5.1 tons/ha (FAO, 2008) with Ethiopia having an average of 3318.03 tons production and 37781.28 hectares planted area (CSA, 2010/11). Among the root crops, taro is perhaps most widely prepared or processed into more consumable forms in the world.

Fresh taro corm has a variation in chemical composition: 63%–85% moisture, 13%–29% starches, 0.60%–1.18% dietary fibers, 1.4%–3.0% proteins, and 0.60%–1.3% ash (Kaushal, Kumar, & Sharma, 2015; Onwueme, 1999). Raw taro contains a considerable amount of oxalic acid (H_2_C_2_O_4_) in the forms of soluble oxalic acid and insoluble oxalate salts (Huang & Tanudjaja, 1992; Kaushal et al., 2015). Soluble oxalic acid can form complexes with calcium, potassium, sodium, and ammonium, and hence reduces mineral availability in the diet and insoluble oxalate salts (i.e., calcium, magnesium, and potassium bind with oxalic acid) cause skin irritation and a pungent odor in unwashed taro corms (Kaushal et al., 2015; Lee, 2002).

Methods of drying affects the properties of the agricultural products such as color, texture, density, porosity, and sorption characteristics of materials (Krokida, Tsami, & Maroulis, 1998). Several drying methods reported in literature such as tray drying, drum drying, and spray drying used in taro flour production are not only unavailable in most developing countries but they are also expensive and require special equipment. In the face of these drawbacks, the use of other available drying methods such as oven, sun, and solar dryer have been considered as better alternatives (Whitfield, 2000).

Different drying methods were reported to produce taro flour (Agoreyo et al., 2011). The methods of drying have been reported to influence chemical composition, for example, reduction in moisture content, calcium oxalate, protein, and lipid, but ash and fiber contents were increased. Taro flour increase the moistness and keeping quality of taro blended bread and high viscosity, high thickening power, and small particle size starch is useful for noodle and bread making (Kaushal et al., 2015; Njintang, Mbofung, & Kesteloot, 2007). In the bread making, low retrogradation tendency of taro flour could reduce the bread stalling, which in turn could increase the shelf‐storage of bread (Taggart, 2004). Despite its nutritional, industrial, and health importance, taro has not gained sufficient research attention to enhance its potential (Aboubakar, Scher, & Mbofung, 2007).

Substitution of taro flour to wheat flour in bread making is an important avenue toward utilization of this crop. This, however, calls for the use of proper flour production methods and suitable taro flour blending ratios through research. The objective of this study was to evaluate the effect of taro drying methods and blending ratios on the physical quality attributes and sensory quality of wheat–taro bread.

## 2 Materials and Methods

### 2.1 Experimental design

A 3^2^ factorial with three replications was used (Table 1). The two factors were wheat–taro flour blending ratio and drying methods; each factor was used at three levels. The upper and lower levels of variables were selected based on different composite to wheat flours studied in the past for bread making (Ikpeme–Emmanuel, Osuchukwu, & Oshiele, 2010; Njintang, Mbofung, Balaam, Kitissou, & Scher, 2008).

**Table 1:** Treatment combinations

Factor 2 (Blending ratio)	Factor 1 (Drying method)		
	D_1_	D_2_	D_3_
B_1_	B_1_D_1_	B_1_D_2_	B_1_D_3_
B_2_	B_2_D_1_	B_2_D_2_	B_2_D_3_
B_3_	B_3_D_1_	B_3_D_2_	B_3_D_3_
Wheat (100%)	Control		

D_1_ = Oven drying, B_1_ = Blending ratio 1 (10 g taro/100 g wheat flour)

D_2_ = Solar drying, B_2_ = Blending ratio 2 (15 g taro/100 g wheat flour)

D_3_ = Sun drying, B_3_ = Blending ratio 3 (20 g taro/100 g wheat flour

C = Control (100 g wheat flour).

### 2.2 Experimental materials

Wheat and taro, both grown in 2010 cropping season, were obtained from Debre Zeit and Areka Agricultural Research Centers, respectively, Ethiopia. The selection criteria of wheat (Kubsa) and taro varieties (Boloso I) were based on bread‐making potential (Habtu, 2010) and bulk production (Adane, 2009), respectively.

### 2.3 Sample preparation

Wheat was milled to particle size of less than 750 μm using the procedure described in the cereal grain processing manual, using the local miller (Bizzarri and Morelli, 1988). Taro roots were, weighed, washed, peeled, sliced (0.6–1.0 cm thick), and soaked in 120 ml lemon juice solution (1/2 cup lemon juice) and 2 L (2.2 quarts) cold water for 45 min to suppress oxidation while they dry (Nelson & Elevitch, 2011). The treated slices were removed, well drained, dehydrated using oven dryer (60°C for 12 hr), solar dryer, and sun drying until moisture reached 14% (Asha and Nair, 2002). The dried taro was milled into flour using a commercial miller. The flour was sieved by 0.75‐mm mesh size sievers and finally packed in air‐tight plastic.

### 2.4 Rheological properties of wheat and taro blended flours

Dough strength was measured by Farinograph (Brabander Farinograph ^®^ E OHG, 2002, Germany) according to AACC (2000) method No.54–21 of constant dough weight method at 30 ± 0.2°C using a 300 g mixing bowl, operating at 63 rpm. Each flour sample in the range of 284.5–300 g on a 14% moisture basis was weighed and placed into the corresponding Farinograph mixing bowl. Water from a burette was added to the flour and mixed to form dough. As the dough was mixed, the farinogram consistence (BU) versus time (min.) was recorded for 20 min. Farinograph values: water absorption capacity (WAC %), dough development time (DT min.), dough stability time (ST min.), mixing tolerance index (MTI FU), time to break down (TBD min.), and farinographic number (FQN FU) were evaluated by AACC Method using the Farinogram software (Brabander^®^ Farinograph version: 2.3.6, 1996–2005, Microsoft corporation).

### 2.5 Bread making

Bread was baked using straight‐dough methods as described in the AACC (2000). It was made with the ingredients (wheat flour [300 g], water [430 g], salt [20 g], sugar [18 g], fat [20 g], and yeast [10 g] and hardened vegetable oil).

### 2.6 Analysis of physical characteristics of bread

#### 2.6.1 Loaf weight, loaf volume, and specific volume

The weight of bread samples were determined after sufficient cooling using a digital balance (0.01 g accuracy) and the loaf volume was determined using rapeseed displacement method (Chopin, 2000) and referred to 100 g of flour on 14% moisture base. The calculation of bread volume was adopted from Sangnark and Noomhorm (2003) as follows:

$$V_{100}=\frac{V_{\text{sr}}}{G}x100$$

Where, V_100_ = Volume calculated for 100 g of the bakery product

V_sr_ = Reading of volume in cm^3.^

G = Weight of one piece of bakery product.

The specific volume of each loaf was calculated as follows:

$$\mathrm{specif}\;\;\mathrm{ic}\;\;\;\text{volume}\;\;\;{\text{(cm}}^{3}\text{/g)}=\frac{\text{Loaf}\;\;\;\text{volume}}{\text{Loaf}\;\;\;\;\text{weight}}$$

#### 2.6.2 Crumb water holding capacity

The bread sample was cut into slices of 1.5 cm thick using a sharp knife. The outer crust of samples was carefully scrapped with kitchen‐type bread knife. The 1 g cuts from each point were combined to make a final weight of about 5 g. The moisture content was determined using connective oven set at 130°C for 1 hr (Shittu, Raji, & Sanni, 2007).

### 2.7 Sensory evaluation

Fifty member judges were selected from staff and graduate students of Haramaya University Department of Food Science and Postharvest Technology. The sensory attributes: visual color, taste, flavors, appearance, and over all acceptability were evaluated using a 5‐point hedonic scale rated from 1 (extremely dislike) to 5 (extremely like). Bread was served at room temperature using the more widely used practice of three digit code during sensory analysis (Resurrection, 1998). Just before each test session, orientation was given to the judges on the procedures of sensory evaluation.

### 2.8 Statistical analysis

The data collected on chemical composition, physical characteristics, and sensory properties were subjected to analysis of variance (ANOVA) with three replications using Statistical Analysis System (SAS, 1990) software version 9.0. Means were compared using Duncan's multiple range test (DMRT) at *p* < .05.

## 3 Results and Discussions

### 3.1 Effect of taro drying methods and blending ratios on physical characteristics of wheat–taro bread

#### 3.1.1 Loaf weight

The results of the effect of blending ratio and drying methods on loaf volume are presented in Table 2. The loaf weight was significantly (*p* < .05) affected by the drying methods, blending ratio and their interaction. The highest was observed in the 20 g and 15 g taro per 100 g wheat flour and the lowest was between the solar‐dried taro flour of 10 g per 100 g wheat flour. In general, with increase in the taro flour, an increase in the loaf weight was observed. Blending ratio appears to be dominant factor compared to the drying methods. Loaf weight is basically determined by the quantity of dough baked gluten functionality and the amount of moisture and carbon dioxide diffused out of the loaf during baking (Shittu et al., 2007). Loaf weight reduction during baking is an undesirable economic quality to the bakers, as consumers often get attracted to bread loaf with higher weight believing that it has more substance for the same price (Shittu et al., 2007).

**Table 2:** Physical characteristics of taro–wheat bread under different drying systems and blending ratio

DM	Loaf weight (g)	Loaf volume (cm^3^)	Specific volume (cm^3^/g)	Crumb moisture (%)
D_1_B_1_	148.81 ± 1.74^bdc^	211.78 ± 20.19^bac^	1.42 ± 0.14^cde^	37.57 ± 0.24^c^
D_1_B_2_	150.62 ± 1.32^bac^	188.02 ± 13.98^bc^	1.24 ± 0.08^de^	40.43 ± 0.43^b^
D_1_B_3_	152.39 ± 1.84^a^	181.67 ± 15.90^c^	1.20 ± 0.11^e^	44.46 ± 0.01^a^
D_2_B_1_	111.65 ± 1.77^f^	237.55 ± 13.83^a^	1.48 ± 0.11^d^	39.43 ± 0.04^b^
D_2_B_2_	114.02 ± 1.67^f^	197.38 ± 13.94^bc^	1.36 ± 0.11^b^	41.15 ± 0.61^b^
D_2_B_3_	117.94 ± 1.98^e^	182.17 ± 17.92^c^	1.30 ± 0.13^cb^	42.06 ± 0.56^b^
D_3_B_1_	146.11 ± 1.60^d^	215.65 ± 19.95^bac^	2.13 ± 0.20 ^cd^	39.60 ± 0.06^b^
D_3_B_2_	147.73 ± 1.56^dc^	202.03 ± 16.30^bc^	1.73 ± 0.15^cde^	40.51 ± 0.39^b^
D_3_B_3_	151.67 ± 1.30^ba^	197.88 ± 12.65^bc^	1.54 ± 0.09^cde^	36.58 ± 0.05^c^
Control	145.93 ± 0.01^d^	240.98 ± 4.18^a^	1.66 ± 0.05^a^	39.35 ± 0.99^b^
Mn	138.69	205.51	1.51	40.09
CV (%)	1.20	6.25	7.21	0.87
*p* < .05	0.0001**	0.0120*	0.0029**	0.0001**

*, **, and ns represent significant at 5%, significant at 1%, and nonsignificant at 5% probability level. Mean values followed by the same letter in the column are not significantly different at 5% probability level. DMRT (*p* < .05), Duncan's multiple range taste at α equal to 0.05; D_1_B_1_, D_2_B_2_, and D_3_B_3_, Oven‐dried taro flour blended bread at 10, 15, and 20 g taro flour, respectively; D_3_B_1_, D_3_B_2_, and D_3_B_3_, Sun‐dried taro flour blended bread at 10, 15, and 20 g taro flour, respectively and D_2_B_1_, D_2_B_2_, and D_2_B_3_, Solar‐dried taro flour blended bread at 10, 15, and 20 g, respectively, Mn, grand mean and CV, coefficient of variance.

#### 3.1.2 Loaf volume

The loaf volume was significantly (*p* < .05) affected by blending ratio and the interaction of drying methods and blending ratio. Drying method had no significant (*p* > .05) influence. The highest was observed for control bread (240.98 cm^3^) and loaf volume decreased with increase in the substitution of taro flour. This is may be due to the gluten protein contents of wheat flour. Lack of the gluten protein contents of taro flour is responsible for the reduction in the loaf volume of leavened taro–wheat flour bread (Belderok, Mesdag, & Donner, 2000; Sidhu, Al‐Hooti, & Al‐Sagar, 1999). Gluten protein contributes the vital role for the increment of loaf volume and elasticity of dough. Loaf volume is affected by the quantity and quality of protein in the flour (Ragaee & Abdel–Aal, 2006).

#### 3.1.3 Specific loaf volume

The results of the effects of drying methods and blending ratio on the specific volume are presented in Table 2. Drying methods, blending ratio and their interaction significantly (*p* < .05) affected the specific volume. As taro flour content increased, the specific loaf volume decreased. This is may be due to the high fiber contents of taro flour that affects the loaf volume of blended bread by diluting the gluten network, which in turn impairs gas retention rather than gas production (Dewettinck et al., 2008; Eiman, Amir, & Mustafa, 2008; Elleuch et al., 2011). The specific loaf volume of bread is the determinate factor for the consumer acceptance. If they are lower than the usual one, consumers are not attracted by it (Shittu et al., 2007).

#### 3.1.4 Crumb moisture

Crumb moisture is the moisture of bread which is found in interior parts of bread, contributes significant effect to estimate the shelf life of bread. The crumb moisture was significantly (*p* < .05) affected by the drying methods, blending ratio and their interaction (Table 2). As the taro flour increased in the blend, the crumb moisture contents also increased. This is probably due to high water binding by starch. As taro flour increased, there is a tendency of moisture to increase, this is probably at large attributed to the high moisture binding nature of small starch granules of taro.

Crumb moisture is important to determine the firmness of fresh bread; if the moisture contents of bread crumb are very high, the firmness of fresh bread is very low (He & Hoseney, 1990; Piazza & Masi, 1995). This result was similar to 40.5%–44.20% and 32%–39% reported by Ognean et al. (2007) and Shittu et al. (2007), respectively.

### 3.2 Rheological properties of wheat and blended flours

#### 3.2.1 Water absorption

**Figure 1:**
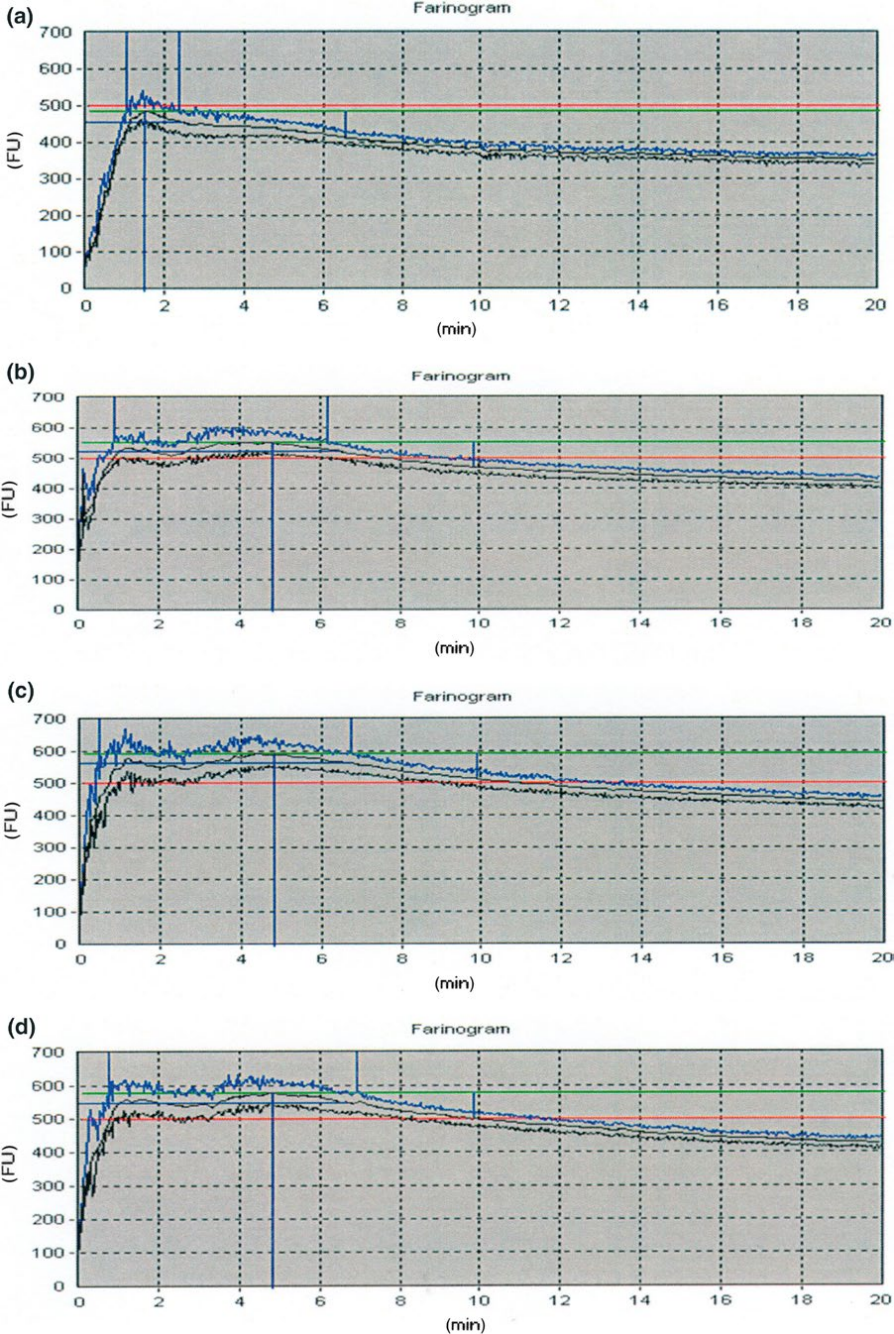
Typical farinograms of wheat (control) (a), blend with 10 g taro flour blend (b), 15 g taro flour (c), and 20 g taro flour (d)

Figure 1 shows the Farinograph curves derived from taro/wheat flour blends. The farinograph properties such as water absorption, dough development time (DDT), stability time (ST), time to break down (TBD)*,* mixing tolerance index (MTI) and farinographic quality number (FQN) were evaluated. The water absorption was significantly (*p* < .05) influenced by the drying method and blending ratio. Water absorption is the point chosen by the baking industry which represents a target water to flour ratio in bread dough. It is important to determine taste, texture, and dough performance during proofing and baking. The WAC plays a major role in the functionality of dough. In particular, WAC has been shown to be related to dough consistency. WAC plays a major role in the functionality of dough. An increase in the taro flour blending ratio resulted in an increased water absorption capacity of blended flour. Such an increasing trend in WAC with increase in taro flour proportion has been reported in earlier studies (Ammar, Hegazy, & Bedeir, 2009; Njintang et al., 2008). The observed increase of WAC could be ascribed to the high level of carbohydrate in taro flour, which was as high as 78%, and is virtually due to the small starch granule size nature with increased surface area of high water absorption capacity (Kaushal et al., 2015). However, an increase in taro flour proportion in wheat–taro composite flour has been reported to decrease the WAC (Ikpeme–Emmanuel et al., 2010). This suggests that other factors such as carbohydrate structure could influence the WAC (Njintang et al., 2008).

#### 3.2.2 Dough development time

Dough development time (DDT) is the time from first addition of water to that of maximum consistency immediately before first indication of weakening. The DDT was significantly (*p* < .05) influenced by blending ratio and the interaction between drying method and blending ratio. There was a general increase in DDT with increase in the taro flour content particularly in sun‐ and solar‐died taro flour. The highest was observed for 20 g/100 g sun‐dried taro flour blended flours. The lowest was observed for control flour (4.00 min.). This might be due to the low gluten protein contents of the blended flours and relatively high amount of bran particles in high extraction rate flours, which may interfere in the quick development of gluten and hydration of endosperm. Dough development time increases with the increase in the proteolytical degradation of protein and with a decrease in the size of starch granule and the increase in the content of damaged starch due to the increase in specific surface area which absorbs water (Thiele, Ganzle, & Vogel, 2002).

#### 3.2.3 Stability time

Stability time is the point between arrival time and departure time and generally indicates the strength of flour (how much gluten flour has and how strong it is). The stability time was significantly (*p* < .01) affected by drying time and the interaction between drying time and blending ratio. There was a general decreasing trend of the stability time with increase in taro flour proportion. The stability time is the gluten quality parameter which describe the viscoelastic properties of formed gluten complex. The stability time of dough is an indicator of the strength, with higher values suggesting stronger dough (Hallen, Ibanoglu, & Ainsworth, 2004; Rossel, Rojas, & Benedito, 2001). A decrease in stability time has been reported in similar study where cow pea flour was used to replace wheat flour. The decrease dough stability time was attributed to relative decrease in the wheat gluten (dilution effect) and completion between wheat protein and cow pea flour protein for water and possible proteolytic activity in the cow pea flour which could possibly has happened in the wheat–taro flour mix (Hallen et al., 2004).

#### 3.2.4 Time to break down

Time to break down is a time from start of mixing until there has been a decrease of 30 FU from peak point. The time to break was significantly (*p* < .01) affected by the drying method and the interaction of drying method and blending ratio. The highest was observed for 15 and 20 g taro flour under sun‐dried taro flour and the lowest was for control flour (7.20 min.). This may be due to the low gluten protein contents of taro flours which take long mixing times to make consistent and uniform blended dough. The difference in the time break down of blended flours due to blending ratio (Table 3) was not significant (*p* > .01) except control flour. This is may be due to the high carbohydrate contents of taro flour and high fiber contents of whole wheat flour. This result is in the range of 2.90–25.4 min reported by Maghirang, Lookhart, Bean, and Pierce (2006) for hard red winter wheat.

**Table 3:** Effect of taro drying methods and blending ratios on rheological properties of wheat and taro blended dough

Blended flours	WA (%)	DDT (min)	ST (min)	TBD (min)	MTI (FU)	FQN (FU)
D_1_B_1_	57.37 ± 0.26 ^cd^	5.20 ± 0.10^dc^	4.80 ± 0.10^d^	9.43 ± 0.31^e^	61.75 ± 1.25^dc^	103.00 ± 1.00^f^
D_1_B_2_	57.60 ± 0.30^bcd^	8.67 ± 0.26^a^	8.03 ± 0.21^a^	13.27 ± 0.29^c^	59.38 ± 2.63^dc^	144.67 ± 3.52^c^
D_1_B_3_	58.40 ± 0.10^a^	5.80 ± 0.10^dc^	7.00 ± 0.26^b^	10.87 ± 0.25^e^	70.00 ± 3.00^ba^	107.00 ± 6.00^f^
D_2_B_1_	57.67 ± 0.26^bc^	5.67 ± 0.26^d^	7.03 ± 0.21^b^	10.57 ± 0.25^e^	76.50 ± 4.50^a^	104.67 ± 2.89^f^
D_2_B_2_	58.20 ± 0.10^a^	8.37 ± 0.26^ba^	6.27 ± 0.06^c^	13.20 ± 0.17^dc^	75.27 ± 2.75^a^	132.33 ± 2.52^d^
D_2_B_3_	58.30 ± 0.20^a^	7.87 ± 0.26^bac^	6.17 ± 0.06^c^	12.07 ± 0.26^d^	76.00 ± 5.00^a^	121.00 ± 3.61^e^
D_3_B_1_	57.10 ± 0.20^d^	6.90 ± 0.20^bc^	8.30 ± 0.20^a^	13.90 ± 0.20^b^	60.38 ± 2.63^dc^	137.67 ± 2.52^d^
D_3_B_2_	57.27 ± 0.15^cd^	8.67 ± 0.16^ba^	8.70 ± 0.10^a^	14.40 ± 0.30^a^	64.00 ± 6.00^bc^	162.00 ± 2.00^a^
D_3_B_3_	58.00 ± 0.66^ba^	9.47 ± 0.16^a^	7.47 ± 0.15^b^	14.63 ± 0.21^a^	56.75 ± 4.25^d^	152.67 ± 2.52^b^
100% wheat	56.03 ± 0.31^e^	4.00 ± 0.72^e^	3.30 ± 0.20^e^	7.20 ± 0.10^f^	75.00 ± 2.00^a^	101.67 ± 1.53^f^
Mn	57.59	7.06	6.71	11.95	67.50	126.67
CV (%)	0.51	2.70	2.34	2.02	2.74	2.49
*p* < .05	0.1854^ns^	0.0001**	0.0001**	0.0001**	0.0001**	0.0001**

** and ns represent significant at 1% and nonsignificant at 5% probability level, respectively. Mean values followed by the same letter in the column are not significantly different at 5% probability level. DMRT (*p* < .05), Duncan's multiple range taste at α equal to 0.05; WA, water absorption; DDT, dough development time; ST, dough stability time; TBD, time to break down; MTI, mixing tolerance index; FQN, farinographic quality number; D_1_B_1_, D_1_B_2_, and D_1_B_3_ are oven‐dried taro flour blended flours at 10, 15, and 20 g taro flour, respectively, D_2_B_1_, D_2_B_2_, and D_2_B_3_ are solar‐dried taro blended flours at 10, 15, and 20 g taro flour, respectively, D_3_B_1_, D_3_B_2_, and D_3_B_3_ are sun‐dried taro flour blended flours at 10, 15, and 20 g, respectively, Mn, grand mean and CV, coefficient of variance.

#### 3.2.5 Mixing tolerance index

Mixing tolerance index is used by bakers to determine the extent that dough will soften over a period of mixing. The mixing tolerance index was significantly (*p* < .01) influenced by drying method, blending ratio and their interaction. Mixing tolerance index (degree of softening) is measured as the distance between the center of the curve at the end of analysis time and the central line which passes through the maximum of the curve. Blending wheat flour with taro had somewhat reduced the mixing tolerance index showing dough stability increased with taro flour addition.

This might be due to the absence of gluten protein contents of taro flour which contributes to the elasticity of dough. As the taro flour blending increased, the mixing tolerance index reduced. It shows taro has improved the dough break down due to over mixing. The degree of softening is the gluten quality parameters which describes the viscoelastic properties of formed gluten complex and increased degree of softening is particularly an important indicator of proteolytic degradation of gluten.

#### 3.2.6 Farinograph quality number

The FQN indicates the quality of flour for bread making. If the flour has poor quality, it gets weakened early and quickly. The drying method, blending ratio and their interaction significantly (*p* < .01) influenced the FQN. An increase in taro flour generally showed an improvement in the FQN. Even though there is such improvement, the bread quality may be not high because this may not necessarily indicate the leavened products produced. The highest the farinographic quality numbers the better dough handling features. Such positive contribution to the blend may be contributed by the high small starch granules in the taro flour.

### 3.3 Sensory characteristics

#### 3.3.1 Color

The sensory scores for color are presented in Table 4. The drying method significantly affected the color of the composite bread. The color difference of taro–wheat bread due to drying methods (Table 4) were significant (*p* < .05). There was no significant (>0.05) difference in color of bread due to blending ratio and the interaction between drying method and blending ratio. However, there was a general decreasing trend in the score with increase in proportion of taro flour dried under solar and the sun. The highest score (4.9, extreme like) was observed for the control sample. Among the experiments, the highest score of 4.6 (like very much) was observed for oven‐ and solar‐dried taro flour blended bread and 4.2 (like moderately) was for sun‐dried taro flour blended bread. The color of bread tells about the appearance of the bread, how it looks like, if it is appealing to the eyes, inviting, and bright. This result was similar to that reported by Sanful (2011) where 100% of the panelists prefer the control (100% wheat) compared wheat–taro flour composite bread. The color difference can be contributed by browning reaction that occurs during drying methods (i.e., via Maillard reaction and caramelization). This color can be due to color of the melanoidin compounds that impart dark color to the crumb of bread. The color change could also be the result of enzymatic browning reaction during the taro processing for flour.

**Table 4:** Effect of drying methods and blending ratio on the sensory characteristics of taro–wheat bread

Bread sample	Color	Taste	Flavor	Appearance	Overall acceptability
D_1_B_1_	4.50 ± 0.06	4.61 ± 0.08^a^	3.55 ± 0.08^ed^	3.26 ± 0.80^d^	4.44 ± 0.07^b^
D_1_B_2_	4.58 ± 0.08	3.75 ± 0.92^bc^	3.36 ± 0.07^ed^	3.97 ± 0.04^b^	4.28 ± 0.04^c^
D_1_B_3_	4.60 ± 0.11	3.28 ± 0.04^c^	3.28 ± 0.13^e^	3.97 ± 0.07^b^	3.95 ± 0.06^d^
D_2_B_1_	4.64 ± 0.16	3.81 ± 0.04^bac^	4.34 ± 0.01^b^	4.09 ± 0.05^ba^	4.55 ± 0.08^ba^
D_2_B_2_	4.40 ± 0.13	4.01 ± 0.08^b^	4.19 ± 0.01^cb^	4.33 ± 0.04^a^	4.50 ± 0.05^ba^
D_2_B_3_	4.44 ± 0.14	3.33 ± 0.06^c^	3.08 ± 0.07^e^	3.75 ± 0.06^bc^	4.19 ± 0.05^c^
D_3_B_1_	4.44 ± 0.08	3.50 ± 0.06^bc^	3.93 ± 0.08^cbd^	3.81 ± 0.05^bc^	4.55 ± 0.08^ba^
D_3_B_2_	3.97 ± 0.05	3.67 ± 0.05^bc^	3.68 ± 0.09^ced^	3.89 ± 0.03^bc^	4.30 ± 0.09^c^
D_3_B_3_	4.27 ± 0.04	3.89 ± 0.04^ba^	3.19 ± 0.04^e^	3.57 ± 0.04^dc^	4.23 ± 0.73^c^
100% wheat	4.97 ± 0.03	4.08 ± 0.07^b^	4.50 ± 0.17^a^	4.35 ± 0.04^a^	4.60 ± 0.09^a^
Mn	4.48	3.79	3.71	3.89	4.36
CV (%)	5.04	7.56	9.43	5.22	5.41
*p* < .05	0.3068^ns^	0.0170*	0.012*	0.002**	0.013*

*, **, and ns represent significant at 5%, significant at 1%, and nonsignificant at 5% probability level, respectively. Mean values followed by the same letter in the column are not significantly different at 5% probability level. DMRT (*p* < .05), Duncan's multiple range taste at α equal to 0.05; D_1_B_1_, D_1_B_2_, and D_1_B_3_ are oven‐dried taro flour blended bread at 10, 15, and 20 g taro flour, respectively, D_2_B_1_, D_2_B_2_, and D_2_B_3_ are solar‐dried taro flour blended bread at 10, 15, and 20 g, respectively, D_3_B_1_, D_3_B_2_, and D_3_B_3_ are sun‐dried taro flour blended bread at 10, 15, and 20 g taro flour, respectively, Mn, ground mean and CV, coefficient of variation.

#### 3.3.2 Taste

The results of the sensory taste scores are presented in Table 4. There was a significant difference (*p* < .05) in the taste of bread due to blending ratio and the interaction between blending ratio and the drying methods. Except taro sun dried, there was a general decrease in the taste score with increase in taro flour proportion. The highest score was 4.6 (close to extremely like) for taro flour dried in oven with taro proportion of 10 g/100 g flour. The least scores were for samples dried under solar and sun with taro proportion of 20 g/100 g. Similar studies reported a decrease in the taste scores of wheat–taro flour composite bread with increased proportion of taro flour (Ammar et al., 2009). This might be due to poor taro flour odor, after taste flavor, and also the high calcium oxalate contents of taro flour which contributes to the salty taste to the blended breads (Kaushal et al., 2015).

#### 3.3.3 Flavor

The flavor of taro–wheat composite bread was significantly (*p* < .05) affected by the drying method, blending ratio and their interaction. Composite bread from taro flour dried under solar dryer with taro flour proportion of 10 g/100 g resulted in the highest score (4.3, moderately like). The flavor scores decrease with increase in taro flour proportion which could be attributed to the high starch contents of taro flour with bland flavor. Flavor is a combination of aroma odor and taste. A decrease in odor and taste score of wheat–taro flour composite bread with increase in taro flour proportion has been reported in earlier studies (Ammar et al., 2009) which is agreement with the findings of this study.

#### 3.3.4 Appearance

Appearance is the surface characteristics of food materials which attracts the consumer perception. The appearance of taro–wheat bread was significantly (*p* < .05) affected by drying method, blending ratio and their interaction. The appearance score for most of the treatment groups was around moderately like. However, composite bread from solar dried taro had higher appearance score whereas the control exhibited the highest appearance score. This might be due to the low gluten protein contents of taro flour which contributes to make less leavened characteristics of blended breads.

#### 3.3.5 Overall acceptability

The overall acceptability scores of wheat–taro composite bread are presented in Table 4. Drying methods and the interaction between drying method and blending ratio significantly (*p* < .05) influenced the overall acceptability. The score ranged from 3.95 to 4.55 which could be associated with like moderately and like very much. However, there was a general decreasing trend in the acceptability score with an increase in the proportion of taro flour. The study revealed that there is possibility of incorporating wheat flour up to 15 g per 100 g of wheat with acceptable sensory attributes of the composite bread. In general, the solar‐dried taro flower resulted in better score in the overall acceptability and other sensory attributes. Similar trend has been reported by Ammar et al. (2009).

## 4 Conclusions

This study showed that physical characteristics, sensory properties of taro–wheat bread, and rheological properties of taro–wheat flour blend dough were significantly affected by drying methods and blending ratio. The acceptability for taro–wheat bread had decreased with increasing taro flour blending ratio due to the presence of salty taste and unusual flavor in the blended bread.

## Conflict of Interest

None declared.
